# Incorporation of a Boron–Nitrogen Covalent Bond Improves the Charge-Transport and Charge-Transfer Characteristics of Organoboron Small-Molecule Acceptors for Organic Solar Cells

**DOI:** 10.3390/molecules28020811

**Published:** 2023-01-13

**Authors:** Jie Yang, Wei-Lu Ding, Quan-Song Li, Ze-Sheng Li

**Affiliations:** 1 Key Laboratory of Cluster Science of Ministry of Education, Beijing Key Laboratory of Photoelectronic/Electrophotonic Conversion Materials, School of Chemistry and Chemical Engineering, Beijing Institute of Technology, Beijing 100081, China; 2 Beijing Key Laboratory of Ionic Liquids Clean Process, CAS Key Laboratory of Green Process and Engineering, State Key Laboratory of Multiphase Complex Systems, Institute of Process Engineering, Chinese Academy of Sciences, Beijing 100190, China

**Keywords:** organoboron, non-fullerene acceptor-based organic solar cells, density functional theory, charge transport, charge transfer

## Abstract

An organoboron small-molecular acceptor (OSMA) M_B←N_ containing a boron–nitrogen coordination bond (B←N) exhibits good light absorption in organic solar cells (OSCs). In this work, based on M_B←N_, OSMA M_B-N_, with the incorporation of a boron–nitrogen covalent bond (B-N), was designed. We have systematically investigated the charge-transport properties and interfacial charge-transfer characteristics of M_B-N_, along with M_B←N_, using the density functional theory (DFT) and the time-dependent density functional theory (TD-DFT). Theoretical calculations show that M_B-N_ can simultaneously boost the open-circuit voltage (from 0.78 V to 0.85 V) and the short-circuit current due to its high-lying lowest unoccupied molecular orbital and the reduced energy gap. Moreover, its large dipole shortens stacking and greatly enhances electron mobility by up to 5.91 × 10^−3^ cm^2^·V^−1^·s^−1^. Notably, the excellent interfacial properties of PTB7-Th/M_B-N_, owing to more charge transfer states generated through the direct excitation process and the intermolecular electric field mechanism, are expected to improve OSCs performance. Together with the excellent properties of M_B-N_, we demonstrate a new OSMA and develop a new organoboron building block with B-N units. The computations also shed light on the structure–property relationships and provide in-depth theoretical guidance for the application of organoboron photovoltaic materials.

## 1. Introduction

Organoboron plays a crucial role in the field of optoelectronic materials applications [1,2,3]. Over the past few years, the emerging strategy of the incorporation of boron-nitrogen (BN) units into organic structures has been widely applied in optoelectronic devices, attracting great attention due to their interesting electronic and optical properties [4,5]. In 2011, Nakamura et al. synthesized a series of BN-fused polycyclic aromatic compounds with high mobility, especially 4b-aza-12b-boradibenzo[g,p]chrysene, which predicted that BN-substituted aromatic hydrocarbons were potential candidates for organic electronic materials [6]. In 2013, Pei and Wang et al. reported two novel tetrathienonaphthalene derivatives incorporating a BN unit, namely BN-TTN-C3 and BN-TTN-C6, signifying that a boron nitride fused ring had been applied to organic electronic devices for the first time [7]. Later, they developed and studied many BN-embedded compounds with excellent properties which could be applied to organic semiconductor materials in the field of electronics [7,8,9]. In 2015, Liu et al. reported the first BN-based acceptor applied in the field of organic solar cells (OSCs) through replacement of a C-C bond by a boron–nitrogen coordination bond (B←N) [10], which sparked great research interest in the chemistry of organoboron acceptors. In 2022, Liu et al. reported an organoboron compound (SBN-1) based on N-B←N units with a balanced resonance hybrid of boron–nitrogen covalent bond (B-N) and B←N, which can be used as an effective building block to construct small band gap conjugated polymers for OSCs [11].

**Figure 1:**
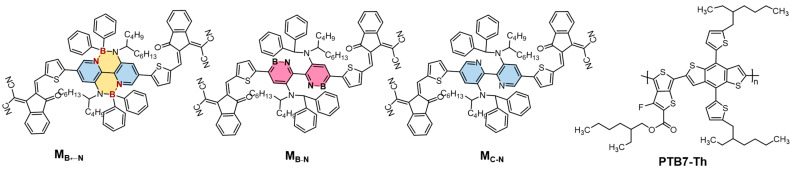
Molecular structures of the investigated acceptors and donor PTB7-Th.

Organoboron provides a new idea to design optoelectronic materials [12,13,14,15,16,17,18,19,20,21,22]. However, the development of n-type organoboron small-molecular acceptors (OSMAs) with advantages of facile synthesis, synthetic versatility, and simplified purification lags far behind that of their polymer counterparts [23,24]. Therefore, it is necessary to further increase the research on OSMAs. The BN unit is one of effective building structures for the construction of OSMAs, which contain two kinds of chemical bonds between B and N, a boron–nitrogen coordination bond, B←N, and a boron–nitrogen covalent bond, B-N. The former has been widely applied to construct non-fullerene acceptors and has achieved great success. The typical representative form is M-BNBP4P-1, which exhibits superior sunlight harvesting capability because of its unique wide absorption spectrum, with two strong bands in the long-wavelength region (771 nm) and the short-wavelength region (502 nm) [25,26]. Besides, Piers et al. devoted their efforts to synthesizing a series of novel cores with BN units for use in optoelectronic devices [27,28]. Nevertheless, the latter is rarely used in OSCs. Recently, Duan et al. developed a novel OSMA called BNTT2F, which was the first reported B-N covalent bond-based electron acceptor for OSCs, achieving the highest power conversion efficiency (PCE) among OSMAs [29]. At this stage, there are not many computational studies regarding OSMAs at the atomistic level, especially concerning their homojunction interfacial charge-transport characteristics and heterojunction interfacial charge-transfer properties [25]. Inspired by the remarkable characteristics of M-BNBP4P-1 (called M_B←N_ in this work) and the fact that the core of the acceptor could effectively regulate its performance, we introduced the B-N bonds into the core group to design a B-N-containing OSMA M_B-N_ and studied whether the derivative containing B-N bond would prove to be an excellent OSMA. The structures of B←N-based OSMA M_B←N_, the B-N-containing compound (M_B-N_), and their carbon-counterpart (M_C-N_) are shown in Figure 1; density functional theory (DFT) and time-dependent density functional theory (TD-DFT) were used to systematically investigate their electronic, optical, and interfacial properties, particularly their charge-transport properties and charge-transfer characteristics. Our results show that the B-N-embedded compound M_B-N_ is regarded as an excellent OSMA, which is expected to improve open-circuit voltage (*V*_OC_), short-circuit current (*J*_SC_), and fill factor (FF) originated from the upshifted frontier molecular orbitals, as well as to exhibit superior charge-transport properties with enhanced electron mobility up to 5.91 × 10^−3^ cm^2^·V^−1^·s^−1^, outstanding optical absorption, and sterling interfacial charge-transfer characteristics. This theoretical work will provide useful guidance for the application of OSMAs in OSCs.

## 2. Results and Discussion

### 2.1. Monomolecular Characteristics

**Figure 2:**
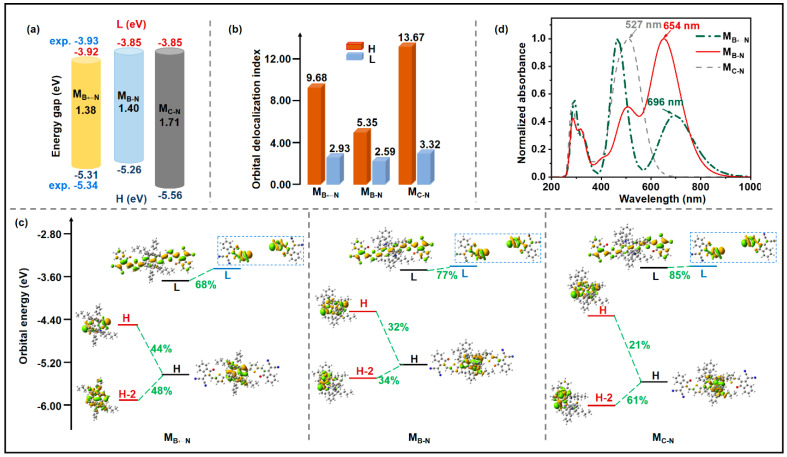
(**a**) Frontier molecular orbital energy levels, (**b**) orbital delocalization index, (**c**) orbital interaction diagram, and (**d**) absorption spectra of M_B←N_, M_B-N,_ and M_C-N_ (H: the highest occupied molecular orbital; L: the lowest unoccupied molecular orbital; green texts indicate the major contribution of the molecular orbitals from the fragments to the orbitals of the complex).

In OSCs, determining the energy-level alignment of an acceptor is particularly important since the key photovoltaic parameter *V*_OC_ is dependent on the difference (Δ*E*_DA_) between the LUMO energy level of the acceptor and the HOMO energy level of the donor (Δ*E*_DA_ = *E*_LUMO_(A)−*E*_HOMO_(D)) [30,31], which is essential to drive devices [32]. As shown in Figure 2a, M_B←N_ and M_B-N_ possess relatively higher-lying HOMO levels than that of M_C-N_, indicating less energy loss (*E*_loss_) in the boron-containing acceptors. Moreover, the empty 2p orbital of the boron atom adopts sp^2^ hybridization in the tri-coordination B-N compounds and sp^3^ hybridization in the tetra-coordination B←N molecules [15,33]. Therefore, the p-π* conjugation with the π system of M_B-N_ due to the empty 2p orbital of the boron atom upshifts the LUMO energy level (−3.85 eV) in comparison with M_B←N_ (−3.92 eV) and then increases Δ*E*_DA_ [33,34,35], which equates to a higher *V*_OC_ in OSCs devices. According to the formula evaluated, *V*_OC_ (e*V*_OC_ = Δ*E*_DA_−*E*_loss_) and the experiment value of M_B←N_ (0.78 V), the *E*_loss_ is estimated as 0.52 eV. Therefore, M_B-N_ yields a high *V*_OC_ of 0.85 V, which is enhanced by about 9% compared to the reference acceptor. The energy gap is related to the planarity of the geometry, and organoboron compounds shows a smaller dihedral angle off the plane. In comparison with M_C-N_, the energy gaps of organoboron compounds greatly decrease because of the substantially upshifted HOMOs; thus, improved absorptions are expected [36]. In terms of M_B-N_, on one hand, its relatively high-lying LUMO levels can result in high *V*_OC_ due to increased Δ*E*_DA_; on the other hand, high *J*_SC_ may be realized because of the reduced energy gaps with upshifted HOMOs compared to those of M_C-N_ [37]. The orbital delocalization index (*ODI*) can quantitatively investigate the degree of orbital delocalization. The smaller the value, the higher the degree of orbit delocalization. As displayed in Figure 2b, the *ODI* values of the LUMOs are smaller than those of the HOMOs, indicating that the studied molecules have good electron transport properties.

Molecular orbital correlation (MOC) analysis has been proven to be an effective method for analyzing the contribution of each molecular orbital fragment to the entire molecular orbital and further exploring the intramolecular orbital interactions [38,39,40]. To study the influence of different forms of boron and nitrogen substitution on the distribution and energy of frontier molecular orbitals, we carried out MOC analysis on these molecules. The studied molecules can be divided into central cores and electron-withdrawing units on both sides. The results (Figure 2c) show that the contributions of the LUMO of the studied molecules are mainly from the electron-withdrawing groups, and the contributions of the HOMO are mainly from the central cores, which explains why the LUMO energy levels of these molecules show little change, but the HOMO exhibits a large shift. Compared with M_C-N_, the introduction of B atoms into the system increases the contribution of the HOMO of the central cores to the whole molecular HOMO, and slightly reduces the contribution of the LUMO of the electron-withdrawing group to the overall LUMO. As an important photovoltaic parameter, the absorbance of materials in OSCs equals the power input, which significantly determines the performance of OSCs [41]. Thus the optical characteristics of molecules are widely impacted [42,43]. As depicted in Figure 2d, the maximum absorption wavelengths of M_B←N_, M_B-N_, and M_C-N_ are calculated as 696 nm, 654 nm, and 527 nm, respectively. The absorption spectra of the organoboron acceptors M_B←N_ and M_B-N_ are red-shifted relative to that of their carbon-counterpart as a result of the reduced energy gaps mentioned previously. M_C-N_ exhibits the strongest absorption at 527 nm, which loses the energy of the long-wavelength region, thus hindering the increase in *J*_SC_. M_B←N_ presents two peaks in the UV-Vis region, which is consistent with previously reported experimental results [25]. Similar to M_B←N_, there are two unique peaks from 400 nm to 800 nm in M_B-N_. Different from M_B←N_, M_B-N_ exhibits stronger absorption at the long wavelength of about 650 nm, while the strongest peak of M_B←N_ is located at 450 nm. In terms of absorption spectra, M_B←N_ and M_B-N_ are expected to be excellent acceptors because they meet the absorption demands of long wavelengths [44,45]. Particularly, M_B-N_ not only exhibits a wide absorption range covering the entire visible region and extending to the near-infrared region with two unique absorption peaks in the visible region, but also presents relatively high absorption intensity, which is expected to improve *J*_SC_ [46].

### 2.2. Acceptor/Acceptor Charge Transport Properties

**Figure 3:**
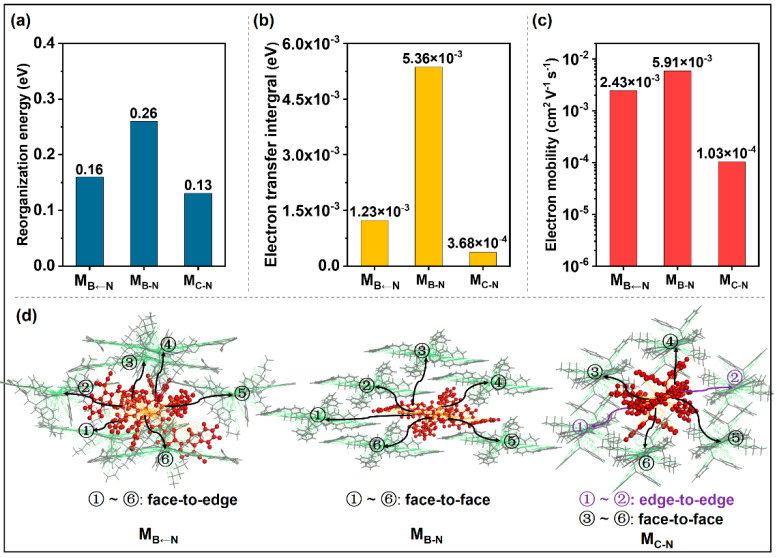
(**a**) Internal reorganization energy (*λ*), (**b**) maximum transfer integral (*v*_max_), (**c**) electron mobility (*μ*_e_), and (**d**) charge hopping pathways of the studied compounds.

Electron mobility (*μ*_e_) is the most important property for acceptors in OSCs, which reflects the electron transport behavior [47,48,49]. In this work, the semi-classical Marcus electron-transfer theory, combined with the Einstein relation (Table S1), was employed to assess *μ*_e_ based on the crystal structures predicted by the polymorph module in Materials Studio [50]. According to the Marcus theory, the one factor affecting the rate is reorganization energy (*λ*): a large *λ* value will lead to a decrease in mobility, subsequently, to poor electron transport [51,52]. As summarized in Figure 3a, introductions of the boron atoms increase *λ* values (0.16 eV for M_B←N_ and 0.26 eV for M_B-N_). The larger *λ* values in organoboron compounds compared with those in M_C-N_ (0.13 eV) indicate larger energy barriers during the electron transfer process [53]. We have to admit that the introductions of boron atoms are disadvantageous in terms of *λ*. The other factor is the electron transfer integral *v* (see Table S1), which reflects the electronic coupling between two neighboring molecules and depends on the relative orientations of adjacent molecules. As shown in Figure 3d, the M_C-N_ molecular stacking includes face-to-face hopping pathways with long distances and edge-to-edge hopping pathways, resulting in relatively small *v* values. Meanwhile, all hopping pathways in M_B←N_ are face-to-edge stacking, but with a small dipole of 0.61 Debye, giving rise to moderate *v* values. The large dipole of the B-N unit is expected to shorten the π-π stacking and construct good molecular packing for the transport charge [54,55,56]. Owing to the large dipole of 3.51 Debye in M_B-N_ due to B-N substitution, the stacking exhibits a compact face-to-face π-stacking pattern, leading to large *v* values and thus, excellent charge transporting properties. As described in the Einstein formula, the maximum electron transfer integral (*v*_max_) plays an important role in *μ*_e_, which is attributed to its large weight in the investigated pathways. It can be seen from Figure 3b that *v*_max_ values of molecules M_B←N_ and M_B-N_ are one order of magnitude larger than those of M_C-N_, with a *v*_max_ of 3.68 × 10^−4^ eV. M_B-N_ has the largest *v*_max_ of 5.36 × 10^−3^ eV, which is about four times that of M_B←N_ (1.23 × 10^−3^ eV, Figure 3a). Interestingly, despite a larger *λ*, M_B-N_ possesses as high as *μ*_e_ of 5.91 × 10^−3^ cm^2^·V^−1^·s^−1^ (Figure 3c), which is one order of magnitude larger than that of M_C-N_ (1.03 × 10^−4^ cm^2^·V^−1^·s^−1^), as a result of its excellent *v*_max_. In addition, M_B←N_ delivers a large *μ*_e_ of 2.43 × 10^−3^ cm^2^·V^−1^·s^−1^, also thanks to its large *v*_max_. In conclusion, although the large *λ* values of M_B←N_ and M_B-N_ are disadvantageous factors, the significant enhancements of *v*_max_ values help to achieve high *μ*_e_ values, especially for B-N-containing M_B-N_ with the largest *μ*_e_, which are conducive to charge transport and thus, are expected to improve the *J*_SC_ and FF parameters of the devices. The results show that *μ*_e_ of organoboron compounds are mainly determined by the *v*_max_ in this work. Although the active layer is amorphous, the small range of the ordered domains of the non-fullerene acceptor is the key to the charge transport characteristics [57]. Here, the information and calculation results are of significance and of reference value.

### 2.3. Donor/Acceptor Interfacial Charge Transfer Performance

In OSCs, the excitons photogenerated within the donor or acceptor components dissociate at the donor/acceptor (D/A) interfaces, which significantly control the photocurrent and thus affect the PCE of the devices [58,59,60,61]. To gain a deeper insight into the interfacial optical properties of the organoboron acceptors, the interfaces between the donor PTB7-Th and the investigated organoboron molecules, namely PTB7-Th/M_B←N_ and PTB7-Th/M_B-N_, were constructed, and the excited-state properties of the D/A interfaces were assessed. These excited states can be divided into three classes, namely, charge-transfer (CT) state, local-excitation (LE) state, and hybrid charge-transfer (HCT) state. Among these, the CT state between the donor and the acceptor at the D/A interface is a paramount intermediate state to realize charge separation [58,62,63], which is characterized by the fact that the two singly occupied molecular orbitals separately locate on the donor and the acceptor. There are generally three mechanisms agreed upon by scientists to form the CT state, namely, the direct excitation mechanism, the intermolecular electric field (IEF) mechanism, and the hot exciton (HE) mechanism [62,64,65]. The LE state refers to an electron excitation localized on the donor or the acceptor, with high transition probability, but which is difficult to separate. The HCT state is another important state which possesses a high exciton utilization, resulting from the CT state, and a large oscillator strength, originating from the LE component; it effectively becomes the CT state in the subsequent process, but only given the relevant mechanisms.

**Figure 4:**
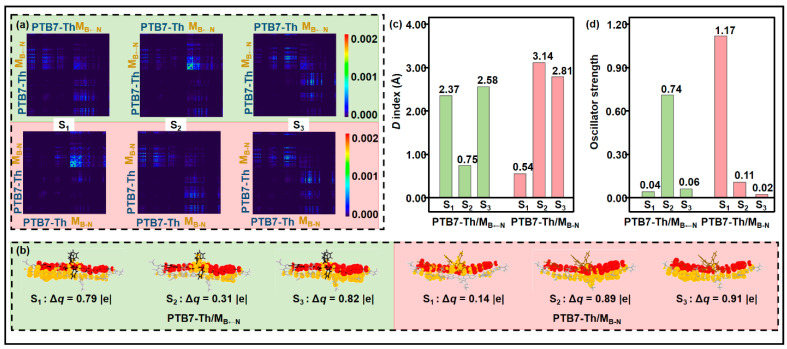
(**a**) 2D site representation of the transition density matrix (TDM), (**b**) charge difference density (CDD) maps with net transferred charge (Δ*q*), (**c**) *D* index, and (**d**) oscillator strengths of the lowest three excited states (S_1_–S_3_) of the studied interfaces (CDD: red/yellow stands for accumulation/depletion of negative charges).

Given that the lower excited states are essential in photo-physical and photochemical processes [66], Figure 4 provides the crucial parameters for the three lowest excited states (S_1_–S_3_), including the transition density matrix (TDM), the charge difference density (CDD) map, the net transferred charges (Δ*q*), *D* index, and the oscillator strength (*f*), which are widely used to evaluate the features of excited states. Among these, the TDM and CDD maps are usually used to visually study the spatial span and primary sites of electron transitions. A large TDM value in the off-diagonal term denotes that a strong electron-hole coherence presents between the donor and the acceptor, which corresponds to the CT state, while a large value in the diagonal region indicates a strong charge coherence within the donor or acceptor, which represents the LE component. Parameter Δ*q* can quantitatively express electrons transferring from the donor to the acceptor [67]. The degree of charge separation can be represented by the *D* index, which is defined as the distance from the hole centroid to the electron centroid [68]. Parameter *f* indicates the transition probability; an excited state with a high *f* value means strong absorption. The direct excitation process is a paramount mechanism to generate a CT state, that is, charge carriers of D/A interface are directly excited into the CT state manifolds upon illumination [66]. According to Figure 4a, the TDM of the first excited state (S_1_) shows that the photoexcitation of PTB7-Th/M_B←N_ is mainly distributed on the off-diagonal part. In addition, as shown in the CDD map (Figure 4b), the electron distributes on the M_B←N_, and the hole distributes on the PTB7-Th; therefore, the S_1_ of PTB7-Th/M_B←N_ is the CT_1_ state. However, the S_1_ of PTB7-Th/M_B-N_ is regarded as the HCT_1_ state, since the photoexcitation of interface PTB7-Th/M_B-N_ distributes not only in the off-diagonal region, but also in the diagonal part. It can be proven by the CDD map, in which the hole only locates in PTB7-Th, and the electron and hole locate in M_B-N_. Note that, in the studied distributions of the second excited state (S_2_), two interfaces are reserved to their S_1_ states, according to the TDM and CDD maps; thus, the S_2_ states are HCT_1_ in the PTB7-Th/M_B←N_ interface and CT_1_ in the PTB7-Th/M_B-N_ interface. PTB7-Th/M_B←N_ and PTB7-Th/M_B-N_ exhibit similar transition characteristics in the third excited states (S_3_), and S_3_ are CT_2_ states in both of the two interfaces. For CT_1_ and CT_2_, the Δ*q* of PTB7-Th/M_B-N_ are larger than those of PTB7-Th/M_B←N_. M_B-N_ transfers more charge to PTB7-Th compared to M_B←N_, reflecting stronger charge-separation ability. The calculated *D* indexes (Figure 4c) follow the order of PTB7-Th/M_B←N_ (2.37 Å) < PTB7-Th/M_B-N_ (3.14 Å) for the CT_1_ states and PTB7-Th/M_B←N_ (2.58 Å) < PTB7-Th/M_B-N_ (2.81 Å) for the CT_2_ states. The larger Δ*q* and *D* index of PTB7-Th/M_B-N_ lead to stronger CT characteristics, which are favorable to the charge separation. In the case of HCT_1_, PTB7-Th/M_B-N_ has the strongest light-harvesting ability, with an *f* of 1.17, which is about 1.5 times larger than that of PTB7-Th/M_B←N_. Our results show that the organoboron M_B-N_ has excellent charge-transfer characteristics of direct excitation into the CT state manifold.

**Figure 5:**
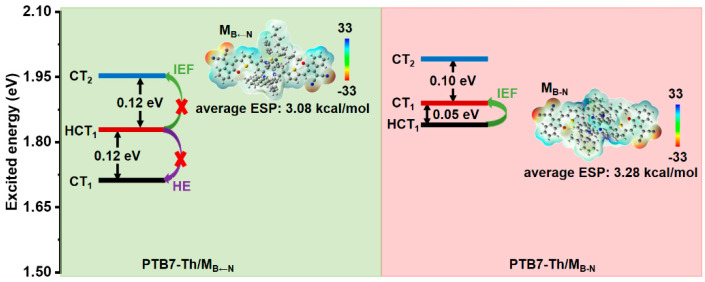
Charge-transfer mechanisms of the lowest three excited states (S_1_–S_3_) for the studied interfaces (IEF: intermolecular electric field mechanism; HE: hot exciton mechanism; ESP: molecular electrostatic potential).

In addition to the interfacial direct excitation mechanism discussed above, the intermolecular electric field (IEF) mechanism is one of the main ways, which separate exciton by producing more CT states [62,69,70]. Two key factors determine the realization of the IEF mechanism, namely energy difference and molecular electrostatic potential (ESP) [71]. The low energy state, with a small energy difference, can generate the CT state through the IEF mechanism driven by the difference in molecular ESP between the donor and the acceptor [72]. The energy difference and average ESP values of these molecules were calculated and summarized in Figure 5. The smaller energy difference of 0.05 eV between the HCT and CT state in PTB7-Th/M_B-N_ can be easier to overcome in comparison with PTB7-Th/M_B←N_, thus promoting interfacial exciton dissociation. In addition, compared to the value of 3.08 kcal/mol in M_B←N_, the average ESP values of M_B-N_ are enhanced to 3.28 kcal/mol. The greatly enhanced ESP of M_B-N_ is favorable for attracting negative electrons and improving charge separation, which is expected to improve *J*_SC_ and FF.

Another frequently discussed mechanism producing the CT state—the hot exciton (HE) mechanism—can generate the CT state from higher-lying states with close energy resonance, which requires the energy difference between adjacent excited states to be as small as possible [43,73,74,75]. For PTB7-Th/M_B←N_ (Figure 5), the energy difference between the high-lying HCT_1_ and the lower CT_1_ state is 0.12 eV, which prevents the production of CT from HCT_1_ through the HE mechanism due to the lack of close energy resonance.

## 3. Computational Methods

### 3.1. Computational Details

The energy minima geometries of the investigated molecules were performed at the B3LYP/6-31G (d, p) level [76], which was confirmed to be suitable for the geometrical parameters [77]. It is well known that the highest occupied molecular orbital (HOMO) eigenvalues are relatively sensitive to the fraction of the Hartree–Fock exchange in the exchange-correlation functional; seven functionals (MPWLYP1M, TPSSH, B3PW91, MPW1B95, PBE38, and M06-2X) with a broad range of Hartree–Fock exchange ratios (from 5% to 54%) were used to calculate the HOMO of M_B←N_ to obtain more accurate HOMO eigenvalues (Figure S1). Note that the computed HOMO energy level using the B3PW91 functional (−5.31 eV) was in very good agreement with the experimental value (−5.34 eV) [25]; thus, functional B3PW91 was chosen to calculate HOMOs in this work. Considering that the virtual orbitals are generally more difficult to describe theoretically than the occupied orbitals, the lowest unoccupied molecular orbital (LUMO) eigenvalues were obtained by adding the corrected HOMO energies to the TD-DFT HOMO-LUMO gap (*E*_1_), namely *E*_LUMO_ = *E*_HOMO_ + *E*_1_ [78]. The molecular packings of the acceptors were obtained from the crystal structure prediction at the DREIDING force field [79], which was considered to be a more appropriate force field for molecular crystal prediction [80]. Crystal structure predictions of the studied molecules were performed by using the polymorph predictor module in Materials Studio [50]. Electrostatic potential charges of all atoms were obtained by the DMol3 module, and the crystal structure prediction was then carried out by employing the Perdew–Burke–Ernzerhof (PBE) [81] exchange-correlation energy functional. Finally, we sorted the obtained crystal structures in terms of their total energies and selected crystal structures with the lowest energies for further DFT calculations regarding their electron mobilities. The M06-2X functional is a high nonlocality functional with a double amount of nonlocal exchange (2X), which provides a good description of the non-covalent interaction [82,83]. Thus, the M06-2X functional was employed to calculate the transfer integrals of all hopping pathways based on the direct coupling approach. To obtain a reliable method, the maximum absorption wavelength of M_B←N_ was calculated by functionals B3LYP, PBE33, PBE38, M06-2X, CAM-B3LYP, and wB97XD (Figure S2). The maximum absorption wavelength (696 nm) obtained by PBE38 is in good agreement with the experimental result of 698 nm [25]. Therefore, the excited-state properties of the investigated compounds were characterized at the TD-PBE38/6-31G (d, p) level. The empirical D3 dispersion corrections were included using the Becke−Johnson damping potential in DFT and TD-DFT calculations [84,85]. The polarizable continuum model was employed in the single molecules to consider the solvation effect in chlorobenzene [86]. The above calculations were performed with the Gaussian 16 code [87]. The large overlap between the donor and the acceptor enhances the interface interactions, thus reducing the overall energy [88]. Therefore, the donor PTB7-Th was stacked face-to-face, stacking M_B←N_ and M_B-N_, respectively, at a distance of 3.5 Å to form the donor/acceptor (D/A) interfaces, which has been proven to obtain essential results [62,89,90]. Then, these D/A interfaces containing a donor/acceptor pair were simulated by molecular dynamics (Figure S3). The equilibrated simulation time was set to 10 ns, with an integration time of 1 fs, using a universal force field [91], which is suitable for organic molecules [92]. The NVE ensemble was performed at 298K using the Forcite module of the Materials Studio software [50]. After reaching equilibrium, the D/A interfacial configuration containing a donor/acceptor pair remained relatively stable, and the lowest energy configurations obtained by equilibrated dynamic simulations were fully optimized at the B3LYP-D3/6-31G (d, p) level with the Gaussian 16 code. Given the slight effect of side chains on the electronic properties, the long alky chains were substituted by the methyl groups in the subsequent computations in order to balance the time and accuracy. The analysis of optical properties was performed using the Multiwfn software [93].

### 3.2. Orbital Delocalization Index

The orbital delocalization index (*ODI*) can quantitatively investigate the degree of orbital delocalization, which is expressed as [93]:

(1) $${ODI}_{i}=0.01\times \sum_{A}{\left(\Theta_{A,i}\right)}^{2},$$

where $\Theta_{A,i}\;$ represents the composition of the A atom in the i orbital.

### 3.3. Electron Mobility

The Marcus theory, with the hopping model, was employed to describe the electron transport behavior [94,95]. The charge hopping rate (*k*) between two identical molecules is [96,97]:

(2) $$k=\frac{2\pi }{h}\upsilon^{2}\frac{1}{\sqrt{4\pi k_{B}T}}\exp\left(\frac{-\lambda }{4k_{B}T}\right)\;,$$

where $k_{B}$, *T*, and *h* are the Boltzmann constant, the temperature in Kelvin, and the Planck constant, respectively (*T* = 300 K in our work). *λ* denotes the reorganization energy, which is calculated using the adiabatic potential energy surface method. In this work, only internal reorganization energy, which mainly originates from the geometrical relaxation during the charge transfer process and reflects the barriers from one molecule to another, was considered. The reorganization energy can be expressed as follows [98]:

(3) $$\lambda =\left(E_{0}^{*}-E_{0}\right)+\left(E_{-}^{*}-E_{-}\right)\;,$$

where *E*_−_^*^ and *E*_0_ are the energies of neutral species in the anionic and neutral geometries, respectively. *E*_0_^*^ and *E*_−_ represent the energies of the anionic species with the geometries of neutral and anionic molecules, respectively.

The transfer integral (*v*) is obtained by adopting a direct approach at the M06-2X/6-31G (d, p) level [30], which has been proven to be suitable in describing the non-covalent interaction. In our work, *v* can be calculated by [99]:

(4) $$\upsilon =〈\Psi_{i}^{\mathrm{LUMO}}|SC\varepsilon C^{-1}|\Psi_{j}^{\mathrm{LUMO}}〉\;,$$

where *Ψ*_i_^LUMO^ and *Ψ*_j_^LUMO^ represent the LUMOs of the isolated molecules i and j. The Kohn–Sham orbital *C* and eigenvalue *ε* are evaluated by diagonalizing the zeroth-order Fock matrix. *S* denotes the overlap matrix for the dimer. The electron mobility of the investigated molecules was calculated using the Einstein relation [99,100]:

(5) $$\mu =\frac{1}{2d}\frac{e}{k_{B}T}\sum r_{i}^{2}k_{i}P_{i}\;,$$

where *d* represents the spatial dimensionality and is 3 in our work, i is a selected hopping pathway, and *r*_i_ and *k*_i_ are the charge hopping centroid-to-centroid distance and charge hopping rate, respectively. *P*_i_ is defined as the hopping probability, which can be obtained using:

(6) $$P_{i}=\frac{k_{i}}{\sum k_{i}}\;,$$

### 3.4. Net Transferred Charge

The net transferred electrons from donor (D) to acceptor (A) can be obtained by using the following formula [67,93]:

(7) $$\Delta q=Q_{D,A}-Q_{A,D}\;,$$

where *Q*_D,A_ (*Q*_A,D_) corresponds to the electron transfer from D (A) to A (D) during the excitation, which can be calculated from:

(8) $$Q_{D,A}=\sum_{i}^{\mathrm{OCC}}\sum_{a}^{\mathrm{vir}}\left[{\left(w_{i}^{a}\right)}^{2}-{\left(w\prime_{i}^{a}\right)}^{2}\right]\sum_{R\in D}\Theta_{R,i}\sum_{S\in A}\Theta_{S,a}\;,$$

where *w*_i_^a^ and *w*_i_^′a^ are the configuration coefficient of the excitation molecular orbital i to a and the de-excitation molecular orbital a to i, respectively; *Θ*_R,i_ (*Θ*_S,a_) is the contribution of atom R (S) to the molecular orbital i (a).

### 3.5. D Index

The distance from the hole centroid to the electron centroid can be expressed from the following equation [68]:

(9) $$D=\sqrt{D_{X}^{2}+D_{Y}^{2}+D_{Z}^{2}}\;,$$

The charge transfer (CT) length in X/Y/Z can be measured by the centroid distances between the hole and the electron in corresponding directions:

(10) $$D_{X/Y/Z}=\left|N_{\mathrm{ele}}-N_{\mathrm{hole}}\right|\;,$$

The electron centroid (*N*_ele_) and hole centroid (*N*_hole_) can be calculated from the following equation:

(11) $$N_{\mathrm{ele}/\mathrm{hole}}=\int n\rho^{\mathrm{ele}/\mathrm{hole}}\left(r\right)dr\;,$$

where *n* is the X (Y or Z) component of position vector **r**. $\rho^{\mathrm{ele}/\mathrm{hole}}$ presents the spatial charge distribution.

## 4. Conclusions

Our studies on two organoboron small-molecular compounds and their carbon counterpart provide an in-depth understanding of the relationship between structures and their electronic, optical, and charge-transport characteristics, as well as their interfacial charge-transfer properties. In comparison with the carbon-counterpart M_C-N_, the introduction of boron strongly lowers the optical gaps and concurrently, dramatically enhances electron mobility due to the unique characteristics originating from the presence of a vacant p orbital on the B atom. The results show that boron atoms are necessary, and that the M_B-N_ containing a boron–nitrogen covalent bond outperforms the M_B←N_ comprising the boron–nitrogen coordination bond due to increased *V*_OC_ (enhanced by about 9% compared to the M_B←N_) as the result of its high-lying LUMO, and its enhanced *J*_SC_ and FF because of excellent absorption and significantly increased electron mobility of up to 5.91×10^−3^ cm^2^·V^−1^·s^−1^. Further, more CT states originating from the direct excitation mechanism and the IEF process help to improve the interfacial charge-transfer properties of PTB7-Th/M_B-N_; thus, M_B-N_-based OSCs are expected to achieve a high *V*_OC_, *J*_SC_, and FF. Our results not only predict an excellent organoboron small-molecular acceptor M_B-N_ by explaining the internal mechanisms, but also provide a theoretical description of the structure–property relationships.

## Data Availability

Data is contained within the article or Supplementary Material.

## Supplementary Materials

The following supporting information can be downloaded at: https://www.mdpi.com/article/10.3390/molecules28020811/s1, Figure S1: The *E*_HOMO_ of M_B←N_ at the different functionals; Figure S2: Maximum absorption wavelength of M_B←N_ employed different functionals; Figure S3: The potential energy evolutions as a function of simulation time; Figure S4: The dihedral angles of studied molecules; Table S1: Electron transfer intervals of the studied molecules; Table S2: Net transferred charge and *D* index of the studied interfaces; Table S3: Charge difference density maps of the studied interfaces.

## Sample Availability

Samples of the compounds are not available from the authors.
