# Supplementary material for: Incorporation of a Boron–Nitrogen Covalent Bond Improves the Charge-Transport and Charge-Transfer Characteristics of Organoboron Small-Molecule Acceptors for Organic Solar Cells
Source: Molecules. 2023 Jan 13;28(2):811. doi: 10.3390/molecules28020811 (PMC9861936; doi:10.3390/molecules28020811)
Supplement: Supplementary file 1 [file molecules-28-00811-s001.zip › molecules-2133870-SI.pdf]

Incorporation of a Boron–Nitrogen Covalent Bond Improves the Charge-Transport and Charge-Transfer Characteristics of Organoboron Small-Molecule Acceptors for Organic Solar Cells

Jie Yang 1, Wei-Lu Ding 2, Quan-Song Li 1,* and Ze-Sheng Li 1,*

1 Key Laboratory of Cluster Science of Ministry of Education, Beijing Key Laboratory of Photoelectronic/Electrophotonic Conversion Materials, School of Chemistry and Chemical Engineering, Beijing Institute of Technology, Beijing 100081, China

2 Beijing Key Laboratory of Ionic Liquids Clean Process, CAS Key Laboratory of Green Process and Engineering, State Key Laboratory of Multiphase Complex Systems, Institute of Process Engineering, Chinese Academy of Sciences, Beijing 100190, China

* Correspondence: liquansong@bit.edu.cn (Q.-S.L.); zeshengli@bit.edu.cn (Z.-S.L.)

Contents

Figure S1 The E_{HOMO} of $M_{B←N}$ at the different functionals……..………....…...…...……S1

Figure S2 Maximum absorption wavelength of $M_{B←N}$ employed different functionals..…S2

Figure S3 The potential energy evolutions as a function of simulation time……...............S3

Figure S4 The dihedral angles of studied molecules…...………....……...……….........…S4

Table S1 Electron transfer intervals of the studied molecules………………................…..S5

Table S2 Net transferred charge and *D* index of the studied interfaces….…………....…......S6

Table S3 Charge difference density maps of the studied interfaces……….........…..…....S7

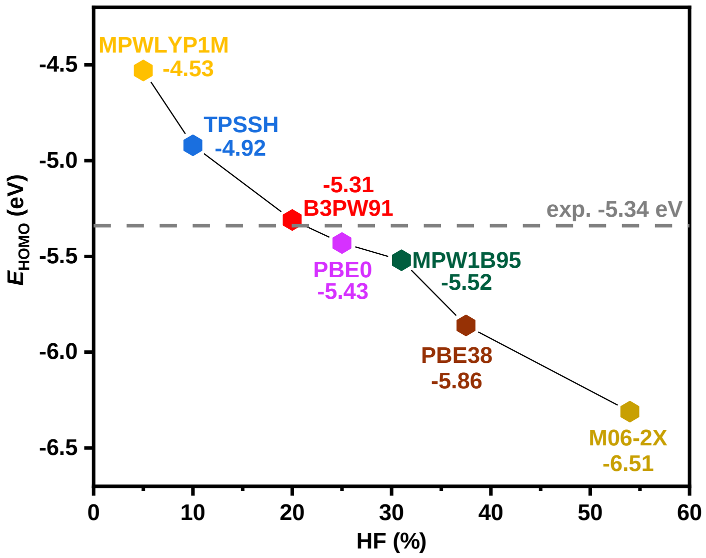

Figure S1. The relationship between the calculated the highest occupied orbital energy level (E_{HOMO}) of $M_{B\leftarrow N}$ and the Hartree-Fock ratio of the employed DFT functionals. The dashed line indicates the experimental value.

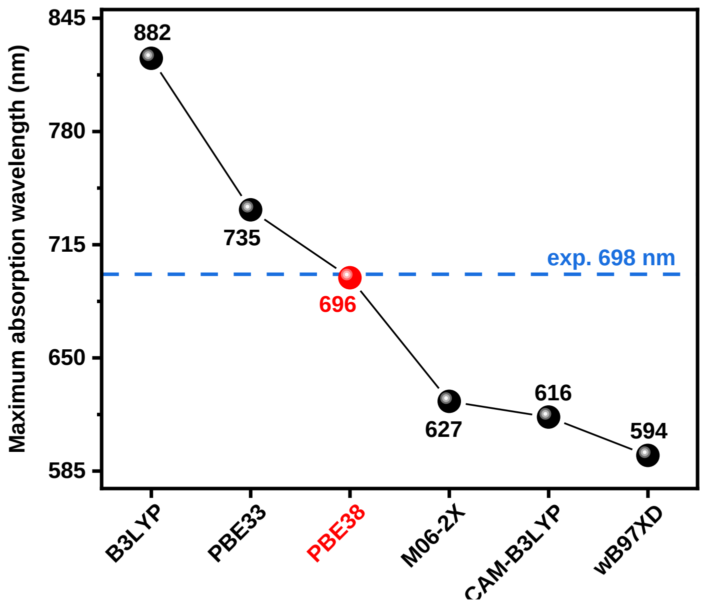

Figure S2. Calculated values of maximum absorption wavelength of $M_{B \leftarrow N}$ employed different DFT functionals along with the experiment value of 698 nm.

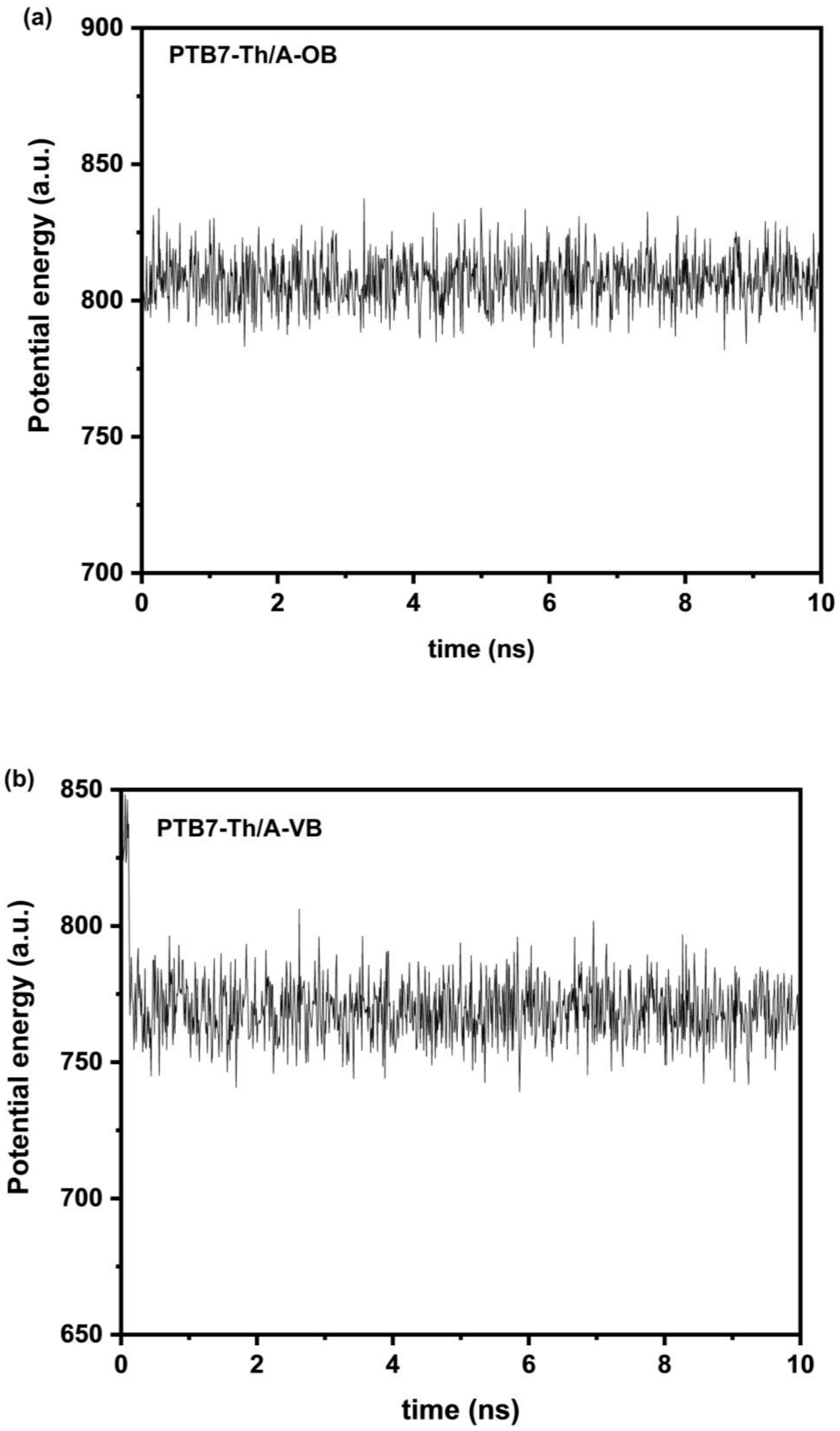

Figure S3. The potential energy evolutions of (a) PTB7-Th/$M_{B\leftarrow N}$ and (b) PTB7-Th/$M_{B\text{-}N}$ as a function of simulation time after equilibration.

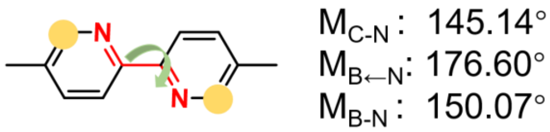

Figure S4. The dihedral angles of studied molecules.

Table S1. The values of electron transfer integral v_i (eV) in the nearest six pathways of the investigated molecules (The bold fonts represent the largest transfer integrals v_{max}).

Path	$M_{B\leftarrow N}$	M_{B-N}	M_{C-N}
v_1	1.68×10^{-4}	2.51×10^{-5}	**3.68×10^{-4}**
v_2	1.67×10^{-4}	2.31×10^{-5}	3.67×10^{-4}
v_3	1.04×10^{-4}	2.50×10^{-6}	4.23×10^{-5}
v_4	1.04×10^{-4}	3.66×10^{-3}	1.09×10^{-4}
v_5	**1.23×10^{-3}**	3.65×10^{-3}	7.13×10^{-5}
v_6	**1.23×10^{-3}**	**5.36×10^{-3}**	4.84×10^{-5}

Table S2. Net transferred charge Δ*q* (|e|) and *D* index (Å) of the lowest sixty excited states of PTB7-Th/$M_{B←N}$ and PTB7-Th/M_{B-N} interfaces.

PTB7-Th/$M_{B←N}$						PTB7-Th/M_{B-N}					
S_n	Δ*q*	*D*	S_n	Δ*q*	*D*	S_n	Δ*q*	*D*	S_n	Δ*q*	*D*
S_1	0.78	2.37	S_{31}	0.62	8.28	S_1	0.14	0.54	S_{31}	0.02	4.34
S_2	0.31	0.75	S_{32}	0.14	2.07	S_2	0.89	3.14	S_{32}	0.83	4.91
S_3	0.81	2.58	S_{33}	0.32	5.39	S_3	0.91	2.81	S_{33}	0.00	0.32
S_4	0.01	0.58	S_{34}	0.57	2.40	S_4	0.01	0.77	S_{34}	0.85	3.13
S_5	0.93	3.07	S_{35}	0.04	3.04	S_5	0.07	0.62	S_{35}	0.04	6.04
S_6	0.48	2.39	S_{36}	0.17	1.30	S_6	0.87	2.98	S_{36}	0.63	2.54
S_7	0.46	2.95	S_{37}	0.42	2.70	S_7	0.90	4.07	S_{37}	0.87	9.72
S_8	0.35	1.70	S_{38}	0.03	1.22	S_8	0.14	0.42	S_{38}	0.23	2.67
S_9	0.48	3.16	S_{39}	0.03	2.36	S_9	0.69	4.82	S_{39}	0.53	2.06
S_{10}	0.09	0.69	S_{40}	0.37	0.86	S_{10}	0.14	2.67	S_{40}	0.80	12.20
S_{11}	0.87	2.83	S_{41}	0.31	2.56	S_{11}	0.79	3.27	S_{41}	0.31	1.13
S_{12}	0.73	2.32	S_{42}	0.25	1.39	S_{12}	0.85	5.95	S_{42}	0.00	1.44
S_{13}	0.74	5.48	S_{43}	0.49	1.22	S_{13}	0.95	3.21	S_{43}	0.36	2.02
S_{14}	0.21	1.98	S_{44}	0.27	2.68	S_{14}	0.07	0.89	S_{44}	0.82	3.49
S_{15}	0.78	2.02	S_{45}	0.76	2.61	S_{15}	0.82	2.44	S_{45}	0.55	4.80
S_{16}	0.06	1.14	S_{46}	0.18	0.61	S_{16}	0.14	1.34	S_{46}	0.58	2.58
S_{17}	0.33	1.03	S_{47}	0.47	4.04	S_{17}	0.06	1.42	S_{47}	0.02	1.26
S_{18}	0.11	0.67	S_{48}	0.35	1.12	S_{18}	0.66	2.72	S_{48}	0.34	3.84
S_{19}	0.09	0.33	S_{49}	0.76	1.98	S_{19}	0.86	4.23	S_{49}	0.70	3.74
S_{20}	0.76	5.04	S_{50}	0.41	1.44	S_{20}	0.26	0.84	S_{50}	0.23	3.10
S_{21}	0.61	2.10	S_{51}	0.60	1.50	S_{21}	0.28	0.96	S_{51}	0.17	3.00
S_{22}	0.24	3.54	S_{52}	0.12	0.94	S_{22}	0.35	1.37	S_{52}	0.53	1.59
S_{23}	0.18	0.64	S_{53}	0.49	0.43	S_{23}	0.50	2.27	S_{53}	0.39	4.52
S_{24}	0.43	1.26	S_{54}	0.32	0.93	S_{24}	0.23	3.07	S_{54}	0.04	1.22
S_{25}	0.17	0.31	S_{55}	0.78	5.29	S_{25}	0.02	1.50	S_{55}	0.12	2.71
S_{26}	0.58	2.01	S_{56}	0.42	1.23	S_{26}	0.41	5.45	S_{56}	0.29	1.45
S_{27}	0.62	1.63	S_{57}	0.50	1.67	S_{27}	0.03	1.89	S_{57}	0.45	2.51
S_{28}	0.04	0.93	S_{58}	0.16	2.39	S_{28}	0.60	3.16	S_{58}	0.56	3.12
S_{29}	0.14	1.77	S_{59}	0.30	1.91	S_{29}	0.14	2.04	S_{59}	0.12	1.39
S_{30}	0.49	1.30	S_{60}	0.41	1.70	S_{30}	0.00	0.89	S_{60}	0.81	2.59

Table S3. Charge difference density (CDD) maps of the lowest sixty excited states of PTB7-Th/$M_{B\leftarrow N}$ and PTB7-Th/$M_{B\text{-}N}$ interfaces.

PTB7-Th/$M_{B\leftarrow N}$						PTB7-Th/$M_{B\text{-}N}$					
S_n	CDD	S_n	CDD	S_n	CDD	S_n	CDD	S_n	CDD	S_n	CDD
S_1	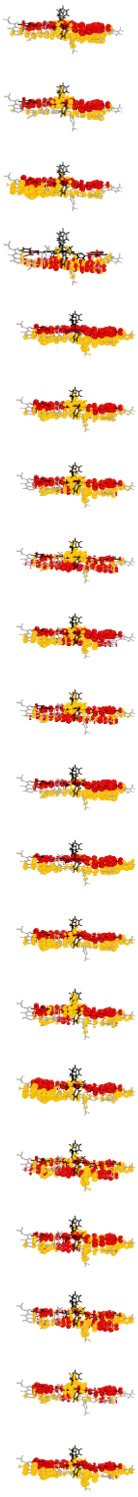	S_{21}	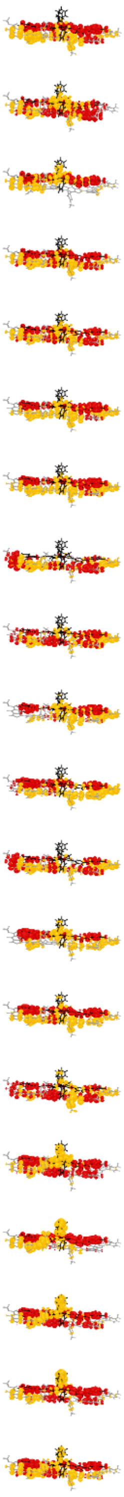	S_{41}	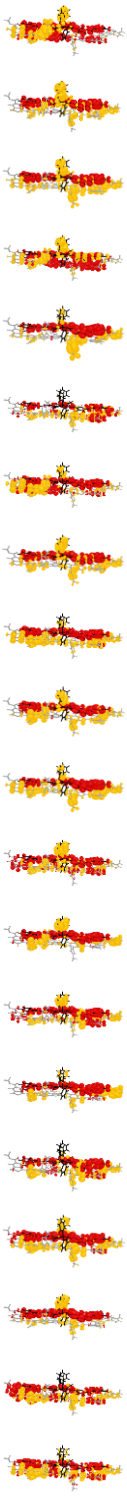	S_1	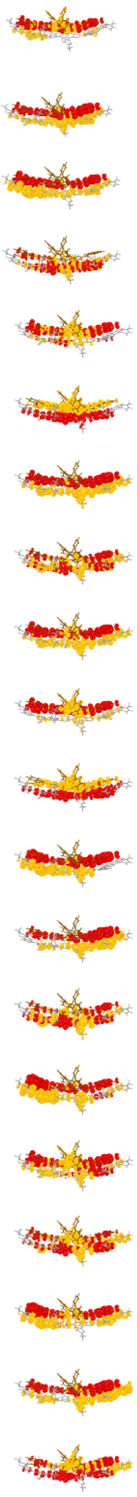	S_{21}	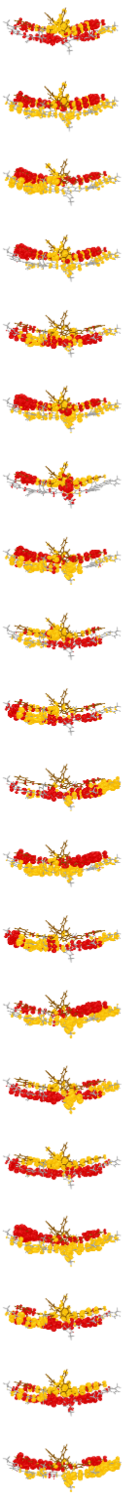	S_{41}	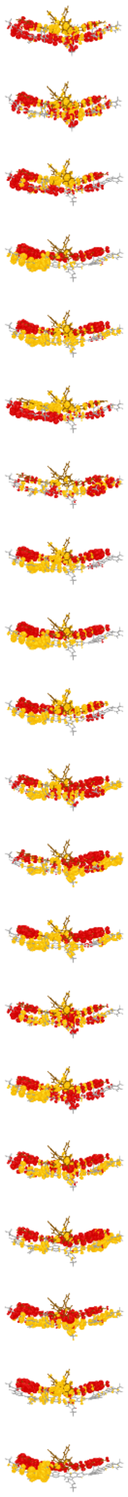
S_2		S_{22}		S_{42}		S_2		S_{22}		S_{42}	
S_3		S_{23}		S_{43}		S_3		S_{23}		S_{43}	
S_4		S_{24}		S_{44}		S_4		S_{24}		S_{44}	
S_5		S_{25}		S_{45}		S_5		S_{25}		S_{45}	
S_6		S_{26}		S_{46}		S_6		S_{26}		S_{46}	
S_7		S_{27}		S_{47}		S_7		S_{27}		S_{47}	
S_8		S_{28}		S_{48}		S_8		S_{28}		S_{48}	
S_9		S_{29}		S_{49}		S_9		S_{29}		S_{49}	
S_{10}		S_{30}		S_{50}		S_{10}		S_{30}		S_{50}	
S_{11}		S_{31}		S_{51}		S_{11}		S_{31}		S_{51}	
S_{12}		S_{32}		S_{52}		S_{12}		S_{32}		S_{52}	
S_{13}		S_{33}		S_{53}		S_{13}		S_{33}		S_{53}	
S_{14}		S_{34}		S_{54}		S_{14}		S_{34}		S_{54}	
S_{15}		S_{35}		S_{55}		S_{15}		S_{35}		S_{55}	
S_{16}		S_{36}		S_{56}		S_{16}		S_{36}		S_{56}	
S_{17}		S_{37}		S_{57}		S_{17}		S_{37}		S_{57}	
S_{18}		S_{38}		S_{58}		S_{18}		S_{38}		S_{58}	
S_{19}		S_{39}		S_{59}		S_{19}		S_{39}		S_{59}	
S_{20}		S_{40}		S_{60}		S_{20}		S_{40}		S_{60}	
